# The Effect of Transfusions after Severe Progressive Hemorrhage

**Published:** 1919

**Authors:** 


***The Effect of Transfusions after Severe Progressive Hemorrhage.*** By W. B. PENFIELD, M. D. (From the Physiological Laboratory, Johns Hopkins University).

At the request of the American Committee on Shock, Medical Division of the National Research Council, a study was made of the relative efficiency of several of the solutions that have been proposed for transfusion in conditions of hemorrhage and shock. This work is limited to a study of hemorrhage. The method adopted was to bring arterial pressure to shock level (40 or 50 mms. Hg.) by successive hemorrhages, and to hold it at this low level by occasional small hemorrhages or transfusions for a determined period varying from 1 to 3 1/2 hours. At the end of the period an intravascular transfusion was made equal in volume to the amount of blood withdrawn. If the animal survived, it was observed during a period of a week. Dogs were used for the experiment and the operations were carried out with aseptic precautions.

The transfusion solutions that were used were —

1) A 0.9 per cent. solution of sodium chloride.

2) The acacia-bicarbonate solution as proposed by Bayliss (gum acacia 6 per cent. plus sodium bicarbonate 2 per cent.)

3) The acacia dextrose solution as proposed by Erlanger and Gasser in their work reported to the Committee on shock. The procedure described by Erlanger and Gasser was not applicable to conditions of hemorrhage. In these experiments, a solution was used containing 6 per cent. of gum acacia and 6 per cent. of dextrose. *Under the conditions described it was found that the gum bicarbonate and the gum dextrose solutions exhibited no advantages over the simple saline solutions.* While the series of experiments was not long, the results seemed decisive regarding the point under investigation. The data obtained in one series are given in the accompanying table.

Observations were made upon the venous pressures throughout the period of experiment, the alkali reserve and the condition of vascular tone as determined by perfusion of an isolated vascular bed (leg). The results will be given in the published paper to appear in the American Journal of Physiology.

TABLE

| N° of Exp. | Transfusion Solution. | Arterial Pressures. | Duration of Low Pressure. | Amount of Hemorrhage in per cent. of Total Blood (calculated). | Final Result. |
|---|---|---|---|---|---|
| V. . . . . . | Na Cl 0.9 %. | 40 mms. | 60 min. | 90 per cent. | Died. |
| VI . . . . . | " | " | 64 " | 93 " | Lived. |
| IX . . . . . | " | " | 75 " | 94.5 " | Lived. |
| X . . . . . | Gum and Bicarbonate. | 40 mms. | 60 min. | 101 per cent. | Lived. |
| IV . . . . . | " | " | 62 " | 101 " | Lived. |
| XII . . . . | " | " | 75 " | 90 " | Died. |
| XI . . . . . | " | " | 89 " | 101 " | Died. |
| XVII . . . . | Gum and Dextrose. | 40 mms. | 62 min. | 90 per cent. | Died. |
| XVIII . . . | " | " | 62 " | 83 " | Died. |
| XVI . . . . | " | " | 63 " | 87 " | Lived. |
| XV. . . . . | " | " | 70 " | 83 " | Died. |

